# Invasive liver abscess syndrome predisposed by *Klebsiella pneumoniae* related prostate abscess in a nondiabetic patient: a case report

**DOI:** 10.1186/s13104-016-2188-y

**Published:** 2016-08-09

**Authors:** Chen-Yi Liao, Ya-Sung Yang, Yen-Cheng Yeh, Ren-Jy Ben, Ching-Chang Lee, Chi-chang Tsai, Chien-Yao Wang, Wu-Hsien Kuo, Chih-Chiang Wang

**Affiliations:** 1 Department of Medicine, Kaohsiung Armed Forces General Hospital, No.2, Zhongzheng 1st Road, Lingya District, Kaohsiung, 802 Taiwan, ROC; 2 Department of Infection, Tri-Service General Hospital, No.325, Sec. 2, Chenggong Road, Neihu District, Taipei, 114 Taiwan, ROC; 3 Department of Nephrology, Tri-Service General Hospital, No.325, Sec. 2, Chenggong Road, Neihu District, Taipei, 114 Taiwan, ROC

**Keywords:** Invasive liver abscess syndrome, *Klebsiella pneumoniae*, Prostate abscess

## Abstract

**Background:**

Prostate abscess is usually a complication of acute urinary tract infection. Invasive liver abscess syndrome is characterized with *Klebsiella pneumoniae* related multiple organ metastasis. Concomitant pyogenic liver abscess and prostate abscess have rarely been reported. Recurrent episode of liver abscess is even rarer.

**Case presentation:**

We report a 71-year-old male with acute bacterial prostate abscess and urinary tract infection caused by *K. pneumoniae* associated with multiple liver abscess, psoas muscle abscess and osteomyelitis. Blood culture and urine culture yielded *K. pneumoniae*, which confirmed the diagnosis of invasive liver abscess syndrome caused by *K. pneumoniae*. The patient was successfully treated with empirical antibiotics for 6 weeks.

**Conclusions:**

This case emphasizes the importance of timely and accurate diagnosis followed by appropriate treatment in disseminated *K. pneumoniae* infection to prevent significant morbidity and mortality.

## Background

Prostate abscess is an uncommon but potentially serious disorder with a mortality rate of 6–30 % before the advent of effective antibiotic therapy. Prostate abscess tends to occur in young and middle-aged men. Entry of the microorganisms into the prostate gland almost always occurs via the urethra. Gram-negative infections, especially with *Enterobacteriaceae* are most common and rarely correlated with *Klebsiella pneumoniae* infection [1]. Clinical symptoms together with an edematous and tender prostate on physical examination should prompt a diagnosis of acute prostatitis. Although most patients with acute prostatitis respond well to antibiotic therapy, a variety of complications can occur, including bacteremia, epididymitis, chronic bacterial prostatitis and prostate abscess. Invasive liver abscess syndrome with concomitant prostate abscess is rare and seldom been reported [2–5]. Herein we present a non diabetic patient with recurrent liver abscess and concomitant prostate abscess predisposed by *K. pneumoniae.*

## Case presentation

A 71-year-old male with past medical history of liver cirrhosis presented with a 5-day history of general malaise, dysuria and lower abdomen fullness. The patient presented with irritative symptoms such as frequency and nocturia. Obstructive symptoms such as poor urine stream, terminal dribbling and incomplete voiding.

**Fig. 1:**
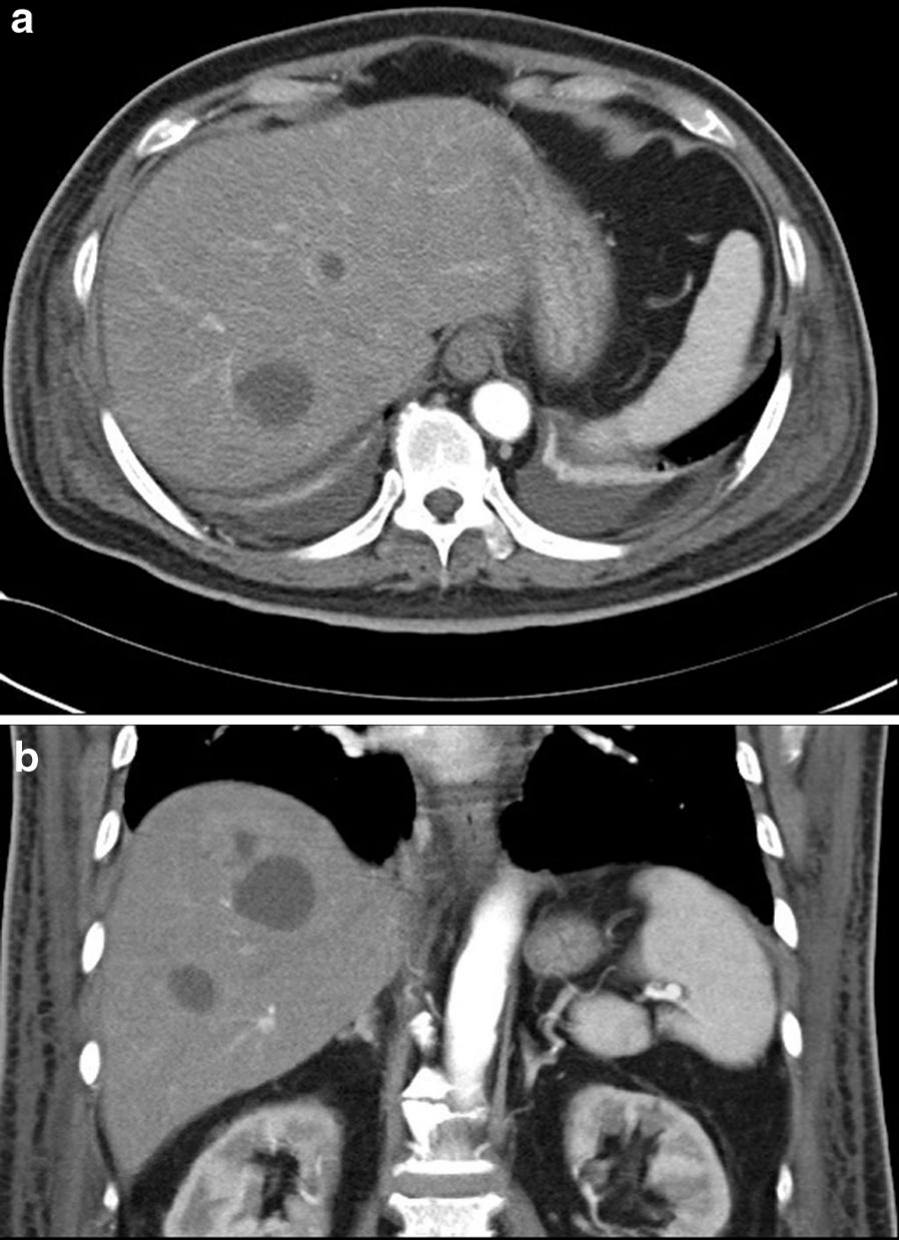
**a**, **b** Contrast-enhanced abdominal CT with contrast denoted numerous lobulated hypodensity lesions in liver compatible with liver abscess overlying segment IV, segment V, segment VI, segment VII, and segment VIII

**Fig. 2:**
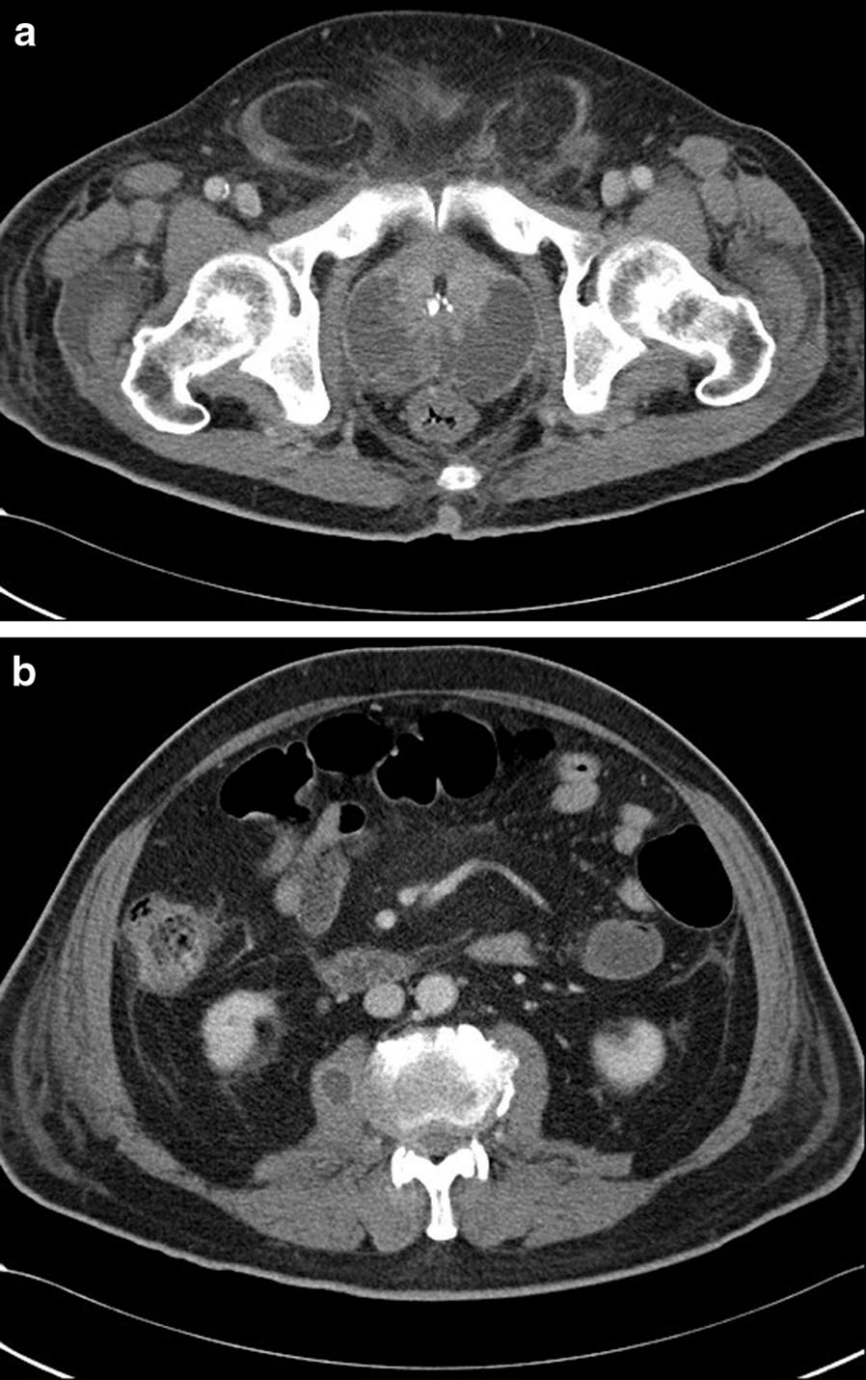
**a** Contrast enhanced abdominal CT depicted prostate abscess with calcification noted in the urethral wall. **b** Contrast enhanced abdominal CT dipicated right psoas muscle abscess

He denied nausea, bowel habit change, body weight loss and fever. He had past liver abscess history with complete resolution 3 years previously. His family history and operation history were unremarkable. On examination, vital signs were stable, and abdominal examination showed unremarkable finding and digital rectal examination showed an extremely tender boggy prostate. Investigations showed, white blood cell (WBC) count of 33,000/μL with band form 15 %, neutrophil 81 %, hemoglobin of 13.9 g/dL, platelet of 51,000/μL, C-reactive protein of 9.62 %, blood urea nitrogen of 86 mg/dL, creatinin of 2.1 mg/dL, total bilirubin of 2.48 mg/dL, direct bilirubin of 0.88 mg/dL, albumin of 2.4 g/dL, AST of 79 U/L, ALT of 64 U/L, alkaline phosphatase of 231 U/L; with PSA total:15.786 ng/mL (0–4), PSA free: 0.255 ng/mL (<0.934), and alpha-feto protein: 1.02 ng/mL (1.09–8.04). HIV and serology for hepatitis B and C were negative. Urine analysis revealed pyuria with urine white blood cells of too numerous to count; blood cultures and urine culture showed growth of *K. pneumoniae*. Chest radiography and KUB revealed unremarkable findings. Abdominal computed tomography demonstrate multiple lobulated liver abscess with a large measurement of about 3.2 × 4 cm without air fluid level. The abscess involved segment IV, segment V, segment VI, segment VII, and segment VIII (Fig. 1). The urinary bladder was thickened secondary to urinary tract infection. The prostate and seminal vesicle was enlarged and hypodense, having fluid density compatible with prostate abscess formation with the right one measuring about 4.3 × 2.4 cm and left one measured about 4.3 × 3.3 cm and seminal vesicle abscess measured about 3.8 × 3.1 cm. Calcification within the urethral wall was noted (Fig. 2a). No evidence of endophthalmitis could be discerned.

**Fig. 3:**
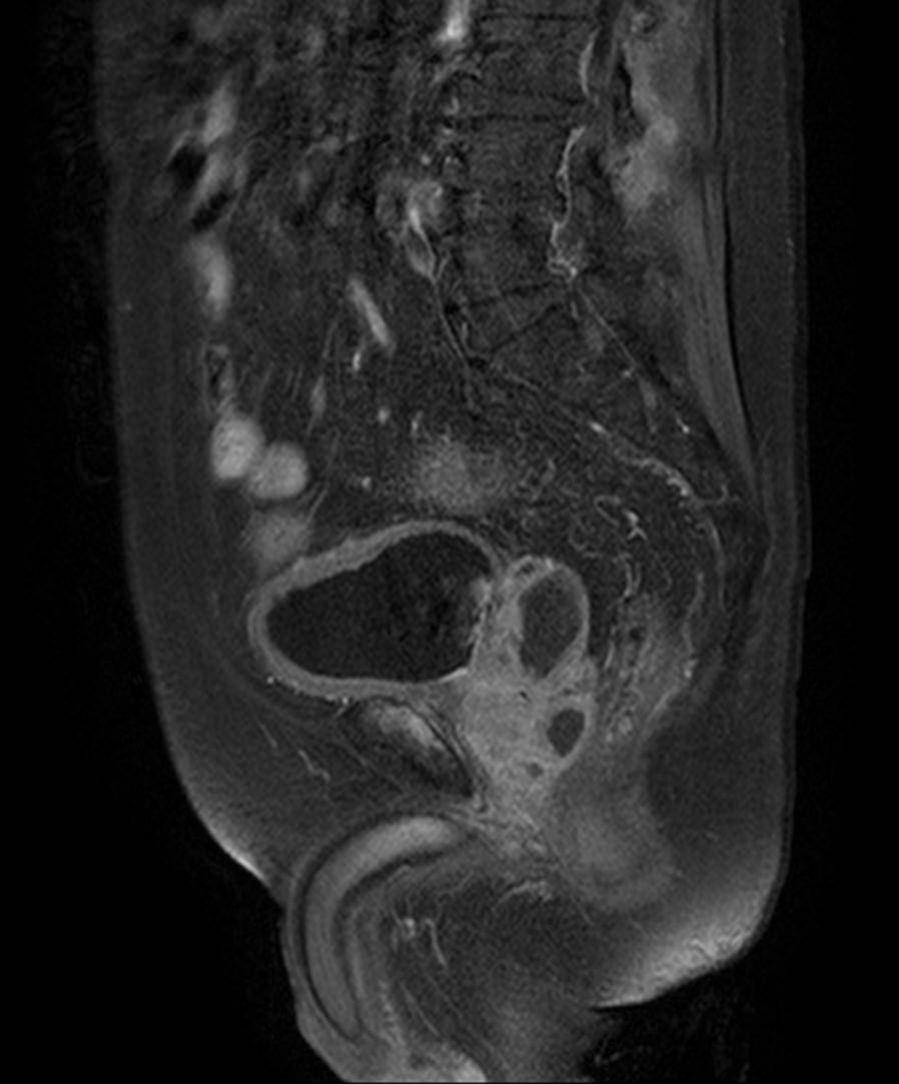
Fat suppressed MRI of pelvis denoted a hyperintensity lesion (*white arrow*) over the right pelvis below the bladder wall compatible with osteomyelietis

The patient was initially started with cefazolin treatment but progressive low back pain, hydrocele and debilitation developed. Repeat contrast enhanced abdominal CT demonstrated progressive liver abscess, prostate abscess and emerging psoas muscle abscess (Fig. 2b). MRI of pelvis demonstrated osteomyelitis over right pubic symphysis (Fig. 3). Antibiotic was shifted to ceftriaxone 2.0 g iv QD for better penetration, and the patient’s clinical condition gradually improved after 6 weeks of empiric antibiotic treatment. The final capsular serotype of *K. pneumoniae* was K1 and genotyping revealed rmpA1, rmpA2 (+) and aerobactin (+).

## Discussion

Invasive liver abscess syndrome is defined by *K. pneumonia*, which is isolated from the abscess aspirate or blood of a patient with imaging findings consistent with a liver abscess in the absence of underlying hepatobiliary disease [6].

In Taiwan, the serotypes K1 and K2 of *K. pneumoniae* are accompanied with presence of the magA and rmpA genes, which contribute to the virulence of hypermuco-viscosity and resistance to in vitro phagocytic uptake by neutrophils as risk factors for metastatic infection [7–10]. Risk factors include patients with impaired host defenses (eg, diabetes mellitus, alcoholism, malignancy, chronic obstructive pulmonary disease, and glucocorticoid therapy). The most common manifestations of metastatic infection are endophthalmitis, meningitis and brain abscess. Other manifestations include lumbar or cervical spondylitis and discitis, septic pulmonary emboli, lung abscess, splenic abscess, necrotizing fasciitis, neck abscess, cerebral abscess, purulent meningitis, otitis media, osteomyelitis, arthritis, prostate abscesses, pylephlebitis and psoas muscle abscesses [11–13].

Diabetes is a frequent underlying illness in reports of *K. pneumoniae* liver abscess, but our patient lacked that putative risk as well. The risk factors in our patient included liver cirrhosis, previous liver abscess and urethral calcification.

Previous liver abscess denote that the liver structure or hepatobiliary system blood flow may be partially destructed which impairs the Kupffer’s cells and renders invasive liver abscess syndrome. Six independent risk factors have been proposed predicting severe complications of *K. pneumoniae* liver abscess including: thrombocytopenia (<100,000/mm^3^), alkaline phosphatase >300 U/L, gas formation in the abscess, APACHE III score >40, use of cefazolin (instead of extended spectrum cephalosporin), and delayed drainage. The initial platelet count in our patient was 51,000/mm^3^ with alkaline phosphatase of 231 U/L, and use of cefazolin initially along with delayed drainage contributed to progression of the invasive liver abscess syndrome.

Relapse of *K. pneumoniae* liver abscess after adequate treatment is rare.

The K1 serotype and rmpA gene in our patient might play important roles as predominant virulent factors which leading to phagocytic resistance [14].

The liver has dual blood supply: sterile arterial blood from the hepatic artery and venous blood from the gut where transient bacteremia of the portal system is not unusual. The high relapse rate of *K. pneumoniae* related liver abscess in our case is probably due to the calcified urethra, which rendered the bacteria colonization.

Except for empiric antibiotic treatment, pigtail catheter drainage is the major treatment strategy for liver abscess unless multiple micro-abscess are present, in which case, fine needle aspiration is satisfactory for both diagnosis and treatment. The patient’s clinical course is usually uneventful if successful pigtail catheter drainage is combined with a 3-week course of parenteral antimicrobial treatment. Pigtail catheter drainage is usually continued for 1–2 weeks, and the drain is removed when culture of the liver abscess become sterile with daily drainage amount <5 mL for several days, and defervescence occurs even after the drainage tube is clamped. Oral antimicrobial treatment for 1–2 months after discharge from the hospital will consolidate the effect of treatment [15]. Our patient declined aggressive intervention and successfully recovered with conservative antibiotic treatment.

## Conclusions

This case emphasizes the importance of timely and accurate diagnosis followed by appropriate treatment in disseminated *K. pneumoniae* infection to prevent significant morbidity and mortality. In patient with impaired host defenses such as diabetes mellitus or alcoholism related liver cirrhosis, we should always keep invasive liver abscess syndrome in mind. Once this syndrome confirmed, various image study for the metastatic lesion, empirical antibiotic use and early adequate drainage of the liver abscess should be undertaken as soon as possible.

## Abbreviations

WBC: white blood cell
AST: aspartate aminotransferase
ALT: alanine aminotransferase
PSA: prostatic specific antigen
HIV: human immunodeficiency virus
KUB: kidney, ureter, bladder
CT: computed tomography
MRI: magnetic resonance imaging
